# Supplementary material for: Heterogeneity of the Immunological and Pathogenic Profiles in Patients Hospitalize Early Versus Late During an Acute Vital Illness as Shown in Native SARS-CoV-2 Infection
Source: Int J Mol Sci. 2025 Mar 6;26(5):2349. doi: 10.3390/ijms26052349 (PMC11900162; doi:10.3390/ijms26052349)

Supplementary

Supplemental Figure S1. There were no differences in alarmin levels over time and in-between clusters.

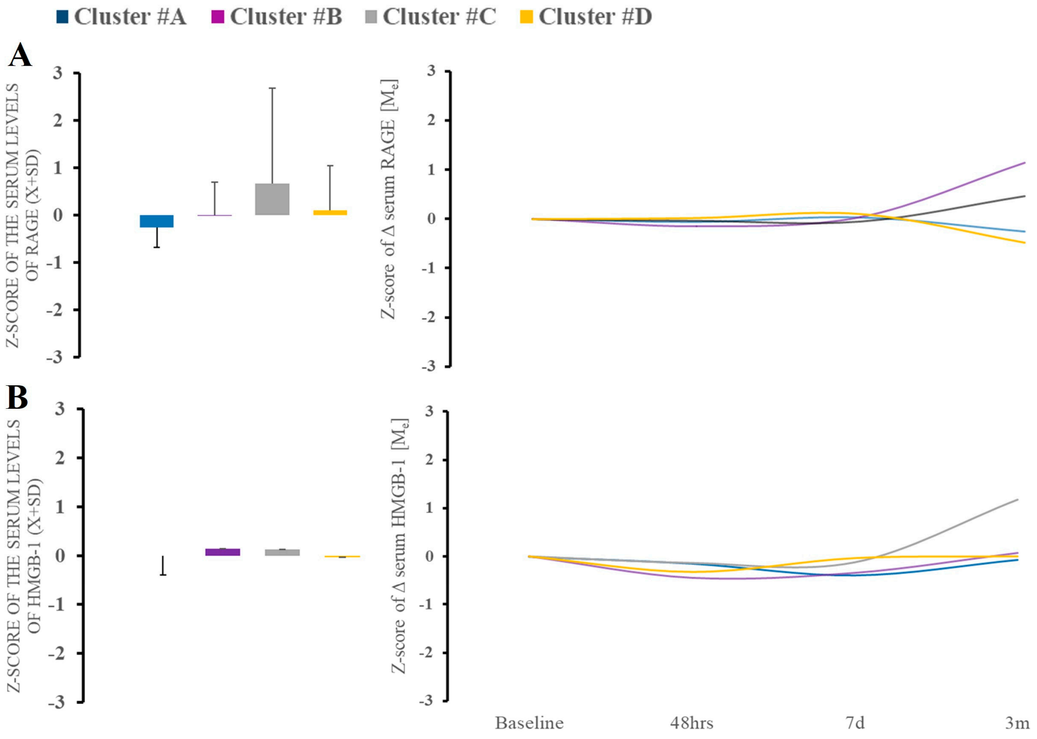

Supplemental Figure S2. There were no differences in several coagulation markers over time and in-between clusters

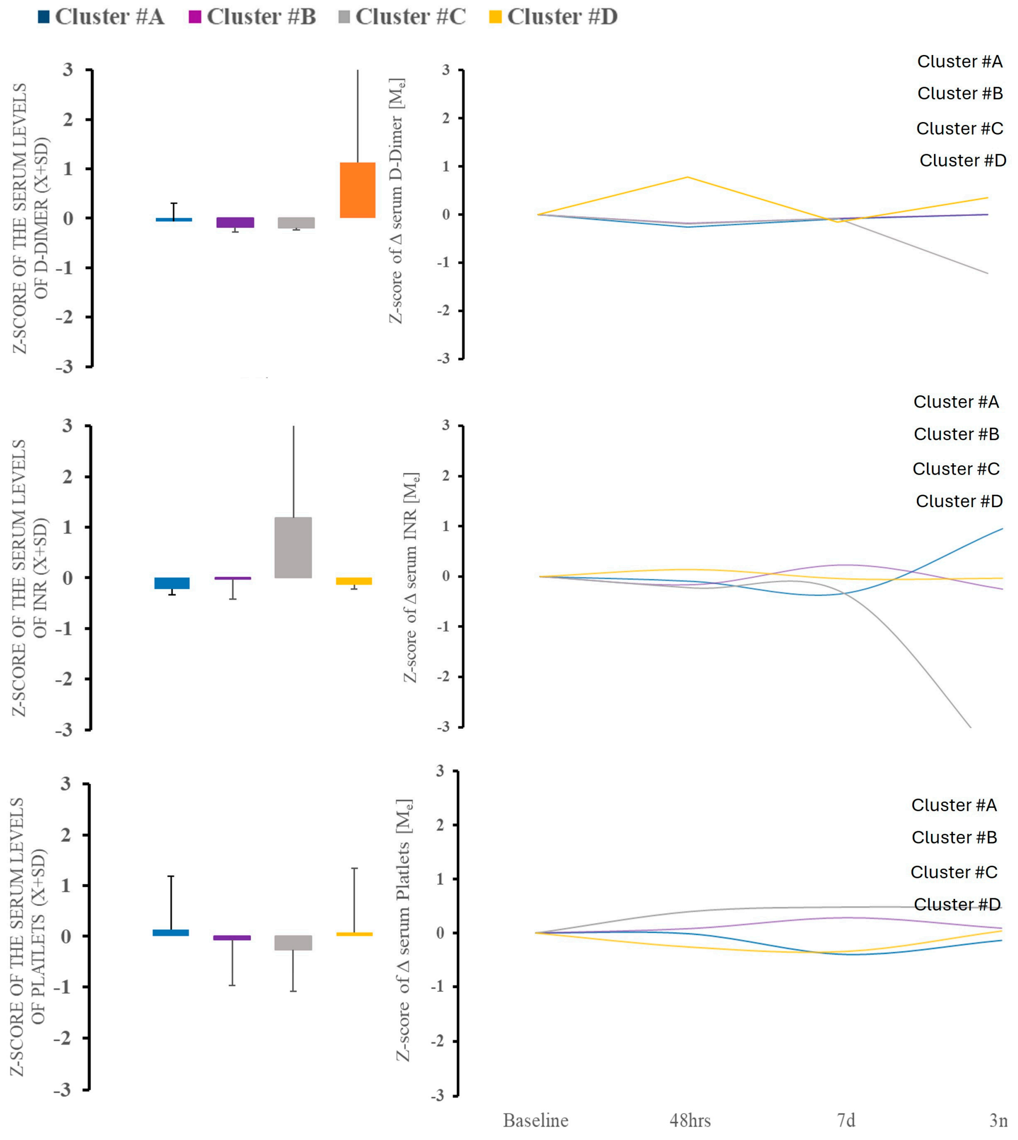

Supplement: Supplementary file 1 [file ijms-26-02349-s001.zip › ijms-3316476-supplementary.pdf]
